# Parental Response to Children’s Chronic Pain

**DOI:** 10.7759/cureus.39149

**Published:** 2023-05-17

**Authors:** Karima Bendahhou, Zineb Serhier, Samir Diouny, Mehdi Simou, Fatima Zahra Mouzoun, Adelin Niyonsaba, Atimad Chemaou, Mohamed Bennani Othmani

**Affiliations:** 1 Epidemiology and Public Health/Cancer, Casablanca Cancer Registry, Ibn Rochd University Hospital, Casablanca, MAR; 2 Laboratory of Medical Informatics, Hassan II University, Casablanca, MAR; 3 Laboratoire Neurosciences Cliniques et Santé Mentale, Hassan II University, Casablanca, MAR; 4 Clinical Neuroscience and Mental Health Laboratory, Faculty of Dentistry, Hassan II University, Casablanca, MAR; 5 Pediatrics, Ibn Rochd University Hospital, Casablanca, MAR; 6 Medical School, Hassan II University, Casablanca, MAR; 7 Clinical Neuroscience and Mental Health Laboratory, Faculty of Medicine and Pharmacy, Hassan II University, Casablanca, MAR

**Keywords:** children, arcs scale, chronic pain, parental behaviors, pediatric pain

## Abstract

Aims: The aim of this study was to describe the behavior of Moroccan parents toward their children's chronic pain.

Methods: A cross-sectional study was conducted in different hospital wards. Parents of hospitalized children with chronic pain aged six or over participated in the study. The parents' behavior toward their children's pain was assessed using an Arabic version of the Adult Responses to Children's Symptoms (ARCS) scale. The scores for each dimension were calculated by summing the responses of the items related to that dimension, and then they were normalized to obtain scores ranging from 0 to 100. The comparison of scores was performed using Student's t-test or ANOVA. The association between quantitative variables was assessed using a correlation coefficient.

Results: A total of 100 parents of children with chronic pain participated in the study. The children's average age was 10.0 ± 2.7 years. The majority of children (62%) experienced pain for more than six months. The joints were the most common location of pain (43%), followed by the abdomen (35%). The "Protect" and "Monitor" dimensions had good reliability with Cronbach's alpha coefficients of 0.80 and 0.69, respectively. The highest mean normalized scores were noted for the "Monitor" and "Protect" dimensions, with means of 82.1 and 70.8, respectively. The "Minimize" dimension had the lowest mean score of 41.4. Parental behavior was not linked to child- or pain-related characteristics. There was no difference in how mothers and fathers behaved towards their children’s pain.

Conclusion: Parents of children with chronic pain in Morocco scored higher on all dimensions of the ARCS, with the highest scores in the "protect" and "monitor" dimensions. These behaviors can negatively affect children's somatic symptoms, functional disability, and anxiety. Our study revealed the importance of providing support to both children and parents of children with chronic pain to manage the pain and related behaviors.

## Introduction

The International Association for the Study of Pain (IASP) defines pain as "an unpleasant sensory and emotional experience associated with actual or potential tissue damage, or described in terms of such damage" [1]. Pain is considered chronic when it persists for a period of three months or more [2]. Several factors may influence the development and chronicization of pain. Some of these factors may include psychological and social factors, such as depression, avoidance behaviors, somatization, and cultural attitudes [3].

Chronic pain prevalence in children and adolescents is estimated to be 46%, increasing with age [4]. This increase in chronic pain may be related to pubertal development, and to the physical, cognitive, emotional, and social changes that occur during adolescence [5]. A meta-analysis on pediatric chronic pain prevalence in low- and middle-income countries reported overall prevalence ranging from 3% to 20%, depending on the location [6].

To effectively understand chronic pain in children, it is important to consider the social and family context in which the pain is experienced [7]. Indeed, parental responses to children's chronic pain complaints play a crucial role in the development and maintenance of children's pain behavior. Caring and protective behaviors have been associated with a variety of negative outcomes in children and adolescents with recurrent/chronic pain [8]. Specifically, parental protective behavior is significantly correlated with child functional disability, and parental minimization is positively linked to pain frequency in children and adolescents with non-cardiac chest pain [8-10]. Additionally, protective behaviors have been found to be associated with a greater risk of child depression, longer duration of pain, frequent school absenteeism, increased symptom maintenance, and greater health care utilization [10]. In addition, protection appears to be closely related to caregivers' own mental state and is positively associated with caregivers' anxiety, depression, and distress and less psychological flexibility and acceptance related to their child's pain [7]. In parallel to protective behavior, which was associated with negative outcomes, such as increased functional disability and longer pain duration, the minimization of pain is believed to cause somatic symptoms, especially in children with high levels of anxiety or depression [8]. Children who experience anxiety or depression often lack effective coping skills when dealing with pain, and parental minimization or criticism may worsen both their physical and emotional symptoms.

The Adult Responses to Children's Symptoms (ARCS) scale was developed to measure how adults respond to children's pain, and its validity and reliability were confirmed in studies of pediatric patients with various chronic pain conditions [10]. Additionally, it has been shown that a five-factor model (Protect, Solicit, Minimize, Monitor, and Distract) for adolescents and a four-factor model (Protect, Minimize, Monitor, and Distract) for children and the combined sample (children and adolescents) have been found to be superior to the initial three-factor model [7]. Therefore, it is important to consider parental environmental behavior in research on pediatric pain, as it may influence the pain trajectory throughout the child's life. To our knowledge, no study in Morocco has explored the behavior of parents with respect to their children's chronic pain. Hence, the significance of this study, which seeks to explore the behavior of Moroccan, parents towards their children's chronic pain.

## Materials and methods

A cross-sectional study was conducted in 2018 in different hospital wards, including the Mother-Child Hospital "Abderrahim El Harouchi" and the Hemato-Oncology Unit of 20 Août University Hospital in Casablanca, Morocco.

Inclusion criteria included the following: children aged six years or over, those with chronic pain persisting for three months or more, and those hospitalized in the selected wards or treated in the daycare unit of the hemato-oncology Unit during the study period. Additionally, those parents of children who met the requirement mentioned above were also present during the study. 

Exclusion criteria included parents and children who refused to take part in the study.

A questionnaire was designed based on the scientific literature to gather information about the age of both parents and children, as well as the educational background and profession of the parents. It also collected data on various pain characteristics, including the duration of the pain, its location, and its intensity, using a visual analogue scale (VAS), frequency during the last week, duration of the last pain attack, and usual schedule of the attack (day/night), as well as analgesics given by parents. Pain intensity was considered mild if VAS was lower than 3, moderate if it is between 3 and 6, and intense if it is above 6. Parents' behavior towards their children's pain was assessed using the Arabic version of the Adult Responses to Children's Symptoms (ARCS) scale. The questionnaire was administered by the investigator to the accompanying parent, while data on pain frequency and intensity were obtained directly from the children.

The ARCS scale was originally developed as an extension of the Illness Behavior Encouragement Scale (IBES) [11], with three dimensions. However, a four-factor model was found to provide a better fit to the data for children and adolescents. Responses were collected using a five-point Likert scale, ranging from "Never" (0) to "Always" (4). To ensure cross-cultural applicability, a 24-item version of the scale, similar to the one used by Noel et al. [7], was adapted following the recommendations of Beaton et al. [12]. Prior to conducting the study, approval was obtained from the head of the department. Parents were informed about the purpose of the study and requested to give their consent. The interviews took place on different days of the week, both in the morning and afternoon, outside of medical visits and care procedures. To ensure the privacy and confidentiality of the children and their families, the questionnaires were anonymous.

The scores for each dimension were calculated by summing the responses of the items related to that dimension, which were then normalized to obtain scores ranging from 0 to 100. Normalized scores of each dimension were obtained by dividing the raw score by the product of the number of items and four and then multiplying by 100. Descriptive statistics, including mean and standard deviation (SD) for quantitative variables and absolute and relative frequencies for qualitative variables were used to summarize the data. The comparison of scores was performed using either Student's t-test or ANOVA. The association between quantitative variables was assessed using a correlation coefficient. The level of significance was set at 5%. SPSS software v.24 (IBM Corp., Armonk, NY) was used.

## Results

A total of 100 parents of children with chronic pain participated in the study. The children's mean age was 10.0 ± 2.7 years. The mean age of parents was 41.0 years (SD=7.4 years), and the majority of accompanying persons were mothers (90%). The mean age of the mothers was 40.6 years (SD=7.2 years), 28% of them were illiterate and 92% were housewives. The fathers had an average age of 44.6 years (SD=8.6 years), with 31% being workers and 9% being civil servants (Table 1).

**Table 1:** Demographic characteristics of children and caregivers (parents) SD: standard deviation

	n (%)
Child age; mean (SD)	10.0 (2.7)
Gender	
Female	51 (51.0)
Male	49 (49.0)
Accompanying person	
Mother	90 (90.0)
Father	10 (10.0)
Parents' age mean (SD)	41.0 (7.4)
Mothers' age; mean (SD)	40.6 (7.2)
Fathers' age; mean (SD)	44.6 (8.6)
Mothers' occupation	
Housewife	92 (92.0)
Government employee	2 (2.0)
Trader	3 (3.0)
Worker	2 (2.0)
Unspecified	1( 1.0)
Fathers' occupation	
Worker	31 (31.0)
Government employee	9 (9.0)
Trader	19 (19.0)
Cab driver	9 (9.0)
Retired / Not working	29 (29.0)
Unspecified	3( 3.0)
Fathers' educational level	
Illiterate	28 (28.0)
Primary	32 (32.0)
Secondary	36 (36.0)
University	4 (4.0)
Mothers' educational level	
Illiterate	57 (57.0)
Primary	27 (27.0)
Secondary	13 (13.0)
University	3 (3.0)

The majority of children (62%) experienced pain for more than six months, while 38% had pain for a period of three to six months. Pain was present for the entire past week in 38% of the children and 35% had pain for four to six days in the week before the study. Most children (65%) experienced both daytime and nighttime pain. The duration of the last attack was between 30 minutes and one hour in 33% of the cases, while it was longer than six hours in 17% of the cases. The joints were the most common location of pain (43%), followed by the abdomen (35%). The mean self-reported pain intensity was 6.3 on a scale of 10, with a SD of 1.6. The pain was moderate in 48% of the children and intense in 47% of the cases. Paracetamol was a widely used analgesic by parents in children with chronic pain, with 60% of parents reporting its use (Table 2).

**Table 2:** Pain characteristics and pain management by parents

	n	Percentage (%)
Etiology		
Juvenile idiopathic arthritis	21	21.0
Acute lymphoblastic leukemia	14	14.0
Systemic lupus erythematosus	10	10.0
Acute myeloid leukemia	9	9.0
Crohn's disease	7	7.0
Behçet's disease	6	6.0
Visceral leishmaniasis	3	3.0
Ewing's sarcoma	3	3.0
Other	27	27.0
Pain lasting for		
3-6 months	38	38.0
6-12 months	60	60.0
>= 12 months	2	2.0
Frequency of pain during the past week		
1 to 3 days	27	27.0
4 to 6 days	35	35.0
all days of the week	38	38.0
Usual timing of the pain crisis		
Diurnal	7	7.0
Nocturnal	28	28.0
Both	65	65.0
Duration of the last painful episode		
< 10 min	3	3.0
[10 - 30[min	23	23.0
[30 - 60[min	33	33.0
[1 - 6[ hours	24	24.0
≥ 6 hours	17	17.0
Localization of pain		
Polyarticular	43	43.0
Abdomen	35	35.0
Extremes (extra-articular)	12	12.0
Hip	5	5.0
Cervical	3	3.0
Diffuse	2	2.0
Pain intensity		
Mild	5	5.0
Moderate	48	48.0
Severe	47	47.0
Management of pain by parents		
Paracetamol	60	60.0
Non-steroidal anti-inflammatory drug	36	36.0
Tramadol	5	5.0

It is important to note that the internal reliability of the Adult Responses to Children's Symptoms (ARCS) scale was satisfactory with a Cronbach's alpha of 0.74. The "Protect" and "Monitor" dimensions had good reliability with Cronbach's alpha coefficients of 0.80 and 0.69, respectively (Table 3). The highest mean standardized scores were noted for the Monitor and Protect dimensions, with means of 82.1 and 70.8, respectively. On the other hand, the "Minimize" dimension had the lowest mean score of 41.4. The items with the highest mean scores were "Check on your child to see how he/she is doing" and "Ask your child questions about how he/she feels" from the Protect dimension and the items "Let your child sleep later than usual in the morning" and "Spend more time than usual with your child" from the Monitor dimension (Table 3).

**Table 3:** ARCS item and dimension scores descriptions. and internal reliability of the scale * Normalized scores were given only for dimensions SD: standard deviation; ARCS scale: Adult Responses to Children's Symptoms scale

Dimension/items	Mean (SD)	Cronbach's alpha
Protect (normalized score*)	70.8 (15.3)	0.8
Do your child’s chores or pick up your child’s things instead of making him/her do it	2.99 (1.11)	-
Get your child something to eat or drink	2.23 (1.19	-
Bring your child special treats or little gifts	1.81 (1.15)	-
Let your child stay home from school	2.93 (1.22)	-
Tell your child that he/she doesn’t have to finish all his/her homework	2.88 (1.12)	-
Give your child special privileges	2.80 (1.11)	-
Stay home from work or come home early	2.73 (1.34)	-
Tell others in the family not to bother your child or to be especially nice to your child	2.56 (1.23)	-
Pay more attention to your child than usual	3.18 (0.91)	-
Let your child sleep in a special place (like in your room or on the couch)	3.18 (1.27)	-
Let your child sleep later than usual in the morning	3.52 (0.88)	-
Keep your child inside the house	2.78 (1.19)	-
Spend more time than usual with your child	3.25 (0.81)	-
Monitor (Normalized score *)	82.1 (16.0)	0.69
Ask your child what you can do to help	2.56 (1.25)	-
Ask your child questions about how he/she feels	3.18 (0.96)	-
Ask your child questions about how he/she feels	3.64 (0.58)	-
Check on your child to see how he/she is doing	3.66 (0.59)	-
Minimize (Normalized score *)	41.4 (15.6)	0.32
Express irritation or frustration with your child	1.38 (1.06)	-
Tell your child there’s nothing you can do about it	1.29 (1.10)	-
Tell your child not to make such a fuss about it	1.25 (1.19)	-
Tell your child that he/she needs to learn to be stronger	2.71 (0.98)	-
Distract (normalized score *)	61.1 (17.1)	0.2
Talk to your child about something else to take your child’s mind off it	2.53 (1.11)	-
Encourage your child to do something he or she enjoys	2.94 (1.08)	-
Try to involve your child in some activity	1.86 (1.13)	-
Overall Cronbach's alpha coefficient		0.74

Parental behavior was not linked to either child or pain-related characteristics. Additionally, there was no difference in how mothers and fathers behaved towards their children’s pain. However, interestingly, mothers with a university education were the least protective with a p-value of p=0.049), while mothers without a professional activity were more protective compared to those with a professional activity (mean Protect score of 72 versus 59, p=0.018), had a higher mean Monitor score (83 versus 68, p=0.009) and minimized less their children's pain with a mean Minimize score of 40.5 versus 52.3 (p=0.038) (Tables 4-5).

**Table 4:** Comparison of ARCS dimension scores according to socio-demographic characteristics of the child and the parents ARCS: Adult Responses to Children's Symptoms; SD: standard deviation

	Protect mean (SD)	p	Monitor mean (SD)	p	Minimize mean (SD)	p	Distract mean (SD)	p
Age	-0.13*	0.201	-0.16*	0.108	-0.16*	0.103	0.038*	0.711
Gender of children		0.724		0.837		0.226		0.294
Female	71.4 (15.8)		81.7 (17.6)		39.6 (15.2)		59.3 (16.8)	
Male	70.3 (14.9)		82.4 (14.2)		43.4 (15.9)		62.9 (17.4)	
Accompanying person		0.106		0.074		0.882		0.714
Mother	71.6 (15.4)		83.1 (15.8)		41.5 (15.4)		60.9 (17.7)	
Father	63.8 (13.1)		73.1 (15.0)		40.6 (18.0)		62.5 (11.9)	
Fathers' educational level		0.833		0.191		0.384		0.500
Illiterate	72.5 (12.8)		77.2 (17.1)		44.0 (16.0)		58.0 (17.2)	
Primary	69.3 (18.1)		86.1 (16.3)		37.1 (17.0)		64.6 (15.4)	
Secondary	70.5 (14.9)		81.8 (14.9)		43.4 (14.2)		60.0 (17.6)	
University	74.5 (14.4)		85.9 (6.0)		40.6 (8.1)		64.6 (26.7)	
Mothers' educational level		0.049		0.387		0.207		0.406
Illiterate	72.0 (14.7)		80.4 (17.5)		42.7 (16.0)		61.7 (16.6)	
Primary	73.3 (13.1)		86.6 (12.5)		38.0 (13.5)		56.8 (18.4)	
Secondary	67.0 (17.3)		81.3 (13.5)		39.4 (16.2)		67.3 (15.0)	
University	43.6 (13.9)		77.1 (23.7)		58.3 (14.4)		61.1 (24.1)	
Mothers' occupation		0.018		0.009		0.038		0.517
Non-active	71.9 (14.9)		83.3 (15.5)		40.5 (15.3)		61.4 (17.2)	
Active	58.7 (15.5)		68.0 (14.7)		52.3 (14.9)		57.3 (16.9)	
Fathers' occupation		0.189		0.293		0.981		0.184
Non-active	74.0 (13.7)		84.7 (17.2)		41.4 (15.9)		64.7 (14.0)	
Active	69.6 (15.8)		81.0 (15.4)		41.5 (15.5)		59.6 (18.1)	

**Table 5:** Comparison of ARCS scores according to characteristics of the child's pain and its origin ARCS: Adult Responses to Children's Symptoms; SD: standard deviation

	Protect mean (SD)	p	Monitor mean (SD)	p	Minimize mean (SD)	p	Distract mean (SD)	p
Causes of pain		0.942		0.181		0.444		0.992
Non-cancerous	71.0 (16.5)		80.6 (15.6)		42.2 (15.5)		61.1 (15.9)	
Cancerous	70.8 (14.9)		85.4 (16.4)		39.6 (15.9)		61.1 (20.0)	
Pain intensity		0.182		0.218		0.576		0.707
Mild	75.4 (12.1)		83.8 (14.4)		45.0 (15.6)		66.7 (15.6)	
Moderate	67.8 (13.8)		78.9 (15.9)		42.8 (13.9)		61.3 (17.9)	
Severe	73.5 (16.6)		85.1 (15.9)		39.6 (17.2)		60.3 (16.7)	
Frequency of pain during the past week		0.089		0.237		0.595		0.153
1 to 3 days	74.8 (12.1)		80.6 (15.5)		44.0 (14.6)		64.5 (15.9)	
4 to 6 days	66.4 (16.9)		79.2 (16.9)		41.4 (17.8)		55.9 (20.3)	
all days of the week	71.6 (15.2)		85.4 (15.3)		40.3 (14.0)		62.9 (14.1)	
Duration of the last painful episode		0.152		0.079		0.068		0.613
10-30 minutes	69.1 (12.9)		73.9 (17.8)		49.5 (16.4)		58.3 (14.4)	
30-60 minutes	67.1 (15.8)		83.0 (13.5)		37.9 (15.1)		63.6 (16.9)	
1-6 hours	76.1 (14.6)		84.1 (18.3)		40.9 (15.7)		60.1 (18.4)	
>6 hours	72.7 (14.6)		86.4 (11.7)		39.3 (12.3)		58.3 (19.5)	
Usual timing of the pain crisis		0.973		0.839		0.692		0.662
Diurnal	72.0 (19.7)		80.4 (16.3)		39.3 (11.8)		61.9 (12.6)	
Nocturnal	70.4 (12.2)		80.8 (16.8)		43.3 (14.4)		63.4 (15.1)	
Both	70.9 (16.2)		82.8 (15.7)		40.9 (16.5)		60.0 (18.4)	

Moreover, a positive and significant correlation was found between the Protect score and the children's pain intensity level (Table 6).

**Table 6:** Correlation between parental behavior and child’s pain characteristics VAS: visual analog scale

		Intensity (VAS)	Frequency	Duration of last episode	Protect	Monitor	Minimize	Distract
Intensity (VAS)	Spearman's rho	—						
	p-value	—						
Frequency	Spearman's rho	0.250	—					
	p-value	0.013	—					
Duration of last episode	Spearman's rho	0.233	0.229	—				
	p-value	0.021	0.024	—				
Protect	Spearman's rho	0.212	-0.043	0.188	—			
	p-value	0.034	0.669	0.065	—			
Monitor	Spearman's rho	0.159	0.144	0.245	0.540	—		
	p-value	0.114	0.154	0.015		—		
Minimize	Spearman's rho	-0.119	-0.095	-0.195	-0.269	-0.310	—	
	p-value	0.237	0.348	0.056	0.007	0.002	—	
Distract	Spearman's rho	-0.025	-0.022	0.021	0.200	0.282	-0.122	—
	p-value	0.804	0.827	0.840	0.046	0.005	0.226	—

## Discussion

The present study explored the behavior of parents toward their children's chronic pain. Results obtained found that 62% of the children experienced pain for more than six months. The majority of children (90%) were accompanied by their mothers. This can be due to the fact that most of the mothers were housewives, unlike fathers who had a professional activity.

To assess parents' responses to their children's pain, the ARCS scale was used. Good internal reliability was observed for the entire scale as well as for two of the four dimensions "Protect" and "Monitor". In their analysis of the factorial validity of the ARCS scale, Noel et al. reported satisfactory Cronbach's alpha coefficients for all dimensions, ranging from 0.63 for "Minimize" to 0.87 for "Protect" in the overall sample including children and adolescents [7].

Our study indicated that the Monitor and Protect dimensions had the highest normalized score averages, with a mean of 82.1 and 70.8, respectively. In contrast, the Minimize dimension had the lowest mean (41.4). The most commonly reported behaviors by parents were related to monitoring with actions such as "Check on your child to see how he/she is doing" and "Ask your child questions about how he/she feels" and protecting with the actions "Let your child sleep later than usual in the morning" and "Spend more time than usual with your child". These results are in line with the findings of Noel et al., who also reported that the highest score was observed for the Monitor dimension (12.85/16) and the lowest score for the Minimize dimension (3.60/20) in both children and adolescents [7]. Likewise, Claar et al. [10] reported a high score on the Monitor dimension including items such as "Check on your child to see how he/she is doing" which had a mean of 3.17 (SD=0.90), "Try to make your child as comfortable as possible" (mean=3.16, SD=0.95) and "Ask your child what you can do to help" (mean=3.09, SD=095). These results are consistent with the results of our study. In contrast, the means of the Protect items in the Claar et al. study were lower than those reported in our study, ranging from 0.80 to 2.31. Similarly, Logan et al. reported a low mean score of 1.38 (SD=0.62) on the Protect dimension [13]. A study conducted in the United States reported racial differences in parental response to their children's chronic pain; Black and Hispanic parents were more protective than White parents with mean scores of 1.93 and 1.65 versus 0.94, respectively [14]. Our study indicated higher means on the Protect dimension than those reported in the literature [8,10,15]. The protective response of parents is known to be associated with functional disability and somatic symptoms in children and adolescents. It is noteworthy that a protective parental response is commonly associated with functional disability and somatic symptoms in children and adolescents.

In fact, the use of protective behaviors by parents can influence a child's pain-related fear and avoidance. Children who are afraid of pain tend to avoid daily activities that could increase their pain, including school attendance. The avoidance of activities can lead to functional disability and somatic symptoms. In their review of the literature, Vlaeyen et al. concluded that decreased daily activity levels were more due to the fear of pain than to pain severity [16]. In our study, we found a positive correlation between protective behavior and children's pain intensity, as well as between monitoring and duration of the last pain episode. Several studies have evaluated the contribution of parental management using cognitive behavioral therapy to reduce the protective response, but the results are controversial [17-19]. In addition, healthcare professionals, including pediatrics, need to be trained to better manage pain in children [20].

There were no significant differences between accompanying mothers' and fathers' behaviors in the present study, regardless of the child's gender, age, or pain characteristics. Hechler et al. reported high levels of catastrophizing among mothers and that maternal catastrophizing was associated with higher levels of child pain intensity [21]. The absence of difference in the behavior of the mothers and fathers in our study could be due to a lack of power since the number of fathers was limited.

However, our study revealed that mothers with a college degree had lower protective behavior ratings (43.6) compared to illiterate mothers and those with primary education, who had a score of 72 and 73, respectively (p=0.049). Working mothers scored less on the Protect and Monitor aspects and scored higher on the Minimize dimension. This could be due to their busy schedule and inability to take time off or leave work early. Therefore, if there is no one to take care of their children, they may not be able to keep them at home during chronic and prolonged pain attacks. This could explain the lower scores observed in the "Protect" and "Monitor" dimensions.

This study has some limitations; the majority of participants were mothers so the description of fathers' behavior was based on a small sample size. Also, the internal reliability of the Minimize and Distract dimensions was low. However, the observed differences in scores were in line with the literature, which supports the good validity of the scale. Further studies should be conducted on a larger sample including more men and exploring the reproducibility of the scale.

## Conclusions

Our study indicated that parents of children with chronic pain in Morocco scored higher on all dimensions of the ARCS, with the highest scores in the "Protect" and "Monitor" dimensions. While these behaviors are important for managing pain and promoting recovery, they can also negatively affect children's somatic symptoms, functional disability, and anxiety; they can impact parents as well. Our study revealed the importance of supporting both children and parents of children with chronic pain to manage their children’s chronic pain and related behaviors. Additionally, it showed the satisfactory psychometric properties of the Moroccan Arabic version of the ARCS, indicating that it can be used as a reliable and valid measure to assess parents' behaviors toward their children's chronic pain in clinical and research settings.
